# Demographic variation in the U.K. serotine bat: filling gaps in knowledge for management

**DOI:** 10.1002/ece3.1174

**Published:** 2014-09-17

**Authors:** Alienor L M Chauvenet, Anthony M Hutson, Graham C Smith, James N Aegerter

**Affiliations:** 1 National Wildlife Management Centre, Animal Health and Veterinary Laboratory AgencySand Hutton, York, YO41 1LZ, U.K; 2 Station Road, Winkfield, Plumpton Green,East Sussex, BN7 3BU, U.K

**Keywords:** *Eptesicus serotinus*, Favourable Conservation Status, prereproductive delay, reproduction, survival, temporal variation

## Abstract

Species of conservation concern, or those in conflict with man, are most efficiently managed with an understanding of their population dynamics. European bats exemplify the need for successful and cost-effective management for both reasons, often simultaneously. Across Europe, bats are protected, and the concept of Favourable Conservation Status (FCS) is used as a key tool for the assessment and licensing of disruptive actions to populations. However, for efficient decision-making, this assessment requires knowledge on the demographic rates and long-term dynamics of populations. We used capture–mark–recapture to describe demographic rates for the Serotine bat (*Eptesicus serotinus*) at two sites in England and investigate the transition rates between three stages: juveniles, immatures, and breeders. We then use these rates in an individual-based population dynamics model to investigate the expected trajectories for both populations. Our results demonstrate for the first time the presence and scale of temporal variation in this species' demography. We describe the lengthy prereproductive period (3.5 years) that female Serotines experience. Finally, we show how site-specific variation in demographic rates can produce divergent population trajectories. Effective management of European bat populations can be achieved through the understanding of life histories, and local demographic rates and population dynamics, in order to anticipate the presence of source and sink sites in the landscape. Using the Serotine bat in England, we show that these can be obtained from rigorous and systematic studies of long-term demographic datasets.

## Introduction

The efficient management of species, either because they cause conflict with man, or because their populations are of conservation concern, is best founded in an understanding of their population dynamics (Beissinger and Westphal 1998). Bats in Europe demonstrate the need for successful and cost-effective management for both reasons, often simultaneously. Populations of many bat species are of conservation concern (e.g., those on annex II of the Habitats Directive 1992). However, bats may also cause problems either directly or indirectly, which need to be resolved within an evidence-based framework because of the legal protections afforded to all species.

Across Europe, bats are protected by national legislation implementing directive 92/43/EEC (Habitats Directive 1992). However, they can cause conflict with humans, either directly by phobic reaction, creating smells, stains, or other damage with feces and urine, with noise or by continual incursion into living and working spaces; or indirectly, where their presence and the legal protections afforded them inhibits human activity or the development of property or adds to their cost. Many species, especially those using anthropogenic roosts, cause such problems. Current threats to bat populations include habitat loss (degrading or removing essential resting and foraging resources), as well as the expansion of land uses which are detrimental to populations, for example urban areas, roads, and wind farms (Walsh and Harris 1996; Hutson et al. 2001; Jones et al. 2003; Stone et al. 2009; Rydell et al. 2010; Berthinussen and Altringham 2012). Synanthropic species face additional pressure from the gradual erosion in the quality and quantity of their roost sites in buildings with increasing redevelopment reducing the quantity and quality of existing roost provision (e.g., Stone et al. 2012 for the U.K.), and ongoing Europe-wide changes in building regulations probably act to limit the rate of creation of new anthropogenic roosts. In addition, across oceanic northern Europe, as seasonal income breeders who rely on concurrent, almost continuous, energy intake to meet the costs of giving birth to usually a single pup, there may be additional pressures on populations caused by an increase in unpredictable and extreme weather events (Jones et al. 2009), which may interrupt foraging and reduce productivity.

The Habitats Directive (1992) uses the concept of Favourable Conservation Status (FCS) as a key tool for the assessment and management of actions disruptive to bat populations (i.e., to resolve conflicts). While poorly defined, at its most simplistic, FCS involves an assessment of the current and likely future state of a population, although demographic rates and population dynamics appear to be rarely used in licensing assessments. The application of quantitative methods and predictive modeling to produce robust (i.e., explicit and transparent) predictions of FCS in European bats is hampered by a general lack of data on demographic rates (Frick et al. 2010) and how they relate to intrinsic and extrinsic roost conditions. As the threats to bats become more diverse and significant, quantitative approaches should be developed to assist decision-making and to improve the successful operation of the directive.

Much effort has been undertaken to measure the ecophysical and landscape properties of roosts to inform the conservation of bats (Catto et al. 1996; Battersby 1999; Harbusch and Racey 2006; Dietz and Pir 2009), which may potentially be used toward the mitigation of disrupted nursery roosts. However, none identified if study sites made a positive contribution to local populations or assessments of FCS. In landscapes of heterogeneous quality, and where national-scale population trends are approximately static (e.g., National Bat Monitoring Programme, NBMP, in Britain), some nurseries will act as population sinks and others as sources and distinguishing between them is important for both decision-making and supporting research into improving mitigation approaches. This information is extremely relevant to FCS and can be gleamed from the robust description of demographic rates in long-lived and spatially dynamic species. However, a comprehensive understanding of demography and population dynamics usually requires capture–mark–recapture (CMR) data collected over decades (Ransome 1989; Frick et al. 2010; Papadatou et al. 2011), which remains rare for the bat taxon.

Such dataset is available for the Serotine bat *Eptesicus serotinus* in Britain. The Serotine bat is a relatively common species across Europe, which is of conservation concern at the northern edge of its range in England, where it has a limited distribution and almost exclusively uses buildings for nursery roosts (Hutson 1991). This brings it into frequent conflict with people where the bats may be unwelcome as they are both relatively large and noisy compared with other British species and droppings are a common source of concern. The species may also be affected by redevelopment. As such, it is a perfect example of a species for which understanding variation in demography and population dynamics is paramount for its conservation and management.

In this study, we describe, for the first time, temporal variation in the demography of the Serotine bat using a robust CMR approach and use population dynamics modeling to predict the dynamics of two communities in Europe.

## Materials and Methods

### Study sites and field methodology

This study collates data from two amateur projects on the Serotine bat in southeastern England: One was a ringing study lead by AMH, in Hollingbury, Brighton, East Sussex, and the other undertaken by the Kent bat group accompanied by AMH in Crundale, Kent. At both sites, annual and ad hoc visits occurred (usually twice between May and August or just once in August), although there are variations in sampling dates and missing data (2 years at Crundale; 11 years at Hollingbury). Here, we present data from 1987 until 2011. The roost at Hollingbury is in the roof void of a former golf clubhouse at the edge of an urban setting (Catto et al. 1996), constructed in *c*. 1909 and largely rebuilt (walls and one chimney retained) in 1990 with provision for the bats (A. M. Hutson, pers. obs.). The roost at Crundale is in the roof void of a family home (built early 1900s), in a rural setting surrounded by small patches of woodland in a pastoral and arable context.

For all visits, the following minimum protocol was applied. Bats were caught using a variety of methods suitable for the site and date. Where bats could be found hanging as a cluster in accessible roof spaces, they were caught by hand, but never extracted from crevices. Capture within roosts was always timed to avoid disturbance to pups and was usually only attempted once pups were flying (i.e., classed as juvenile). On other occasions, bats were caught outside the roost using static hand nets or harp traps. At neither site can it be assumed that all of the bats present were captured on any occasion, although the proportion regularly caught at Crundale was probably higher than at Hollingbury. Each bat was described, recording species, sex, age (juvenile or adult based on ossification of the metacarpal epiphyses), current reproductive status (with breeding confirmed by palpation of the abdomen or the presence of distended nipples; Racey 1988), length of forearm (to 0.1 mm, almost always measured by AMH), and weight (to 0.1 g). In addition, a uniquely numbered alloy ring (4.2 mm, The Mammal Society) was used to mark each bat. All capture, handling, and marking were under license from Natural England or its predecessors.

### Analysis

We used a multistate capture–mark–recapture model in MARK (Cooch and White 2013) with uneven time intervals (run in R-v3.0.0; R Core Development Team 2013). Due to the poor record of male recapture (0 at Hollingbury; 1 at Crundale), males were excluded from the analysis. For both sites, individual captures were assigned to one of three life stages, juveniles (J), immatures/unknowns (U), and breeders (B). The U stage was assigned to bats appearing to be adult but where breeding was not recorded or could not be determined; this could potentially include immature bats, reproductively senescent bats, mature bats not breeding that year (nonbreeding breeders), or bats caught on dates when breeding could not be assessed.

Using multistate models, female survival rates (*S*), recapture probabilities (*p*), and transition probabilities between stages (*ψ*) were estimated. Multistate models can produce poor parameter estimates for small datasets such as this (Schaub et al. 2004), necessitating the limited number of stages. Moreover, because all transitions between states were not possible or considered unlikely, some probabilities were fixed to 0: *ψ*_J→J_, *ψ*_B→J,_ *ψ*_B→U._ While in principle mature bats might breed intermittently (i.e., *ψ*_B→U_ ≠ 0), we assumed that all bats found to have bred once had entered the “breeder” state and remained so regardless of their reproductive output the following years. This assumption was made because (1) the data were insufficient to support an additional state in MARK, and the loss of uncertain observations would significantly limit its analysis, and (2) congeners show robust evidence of attempting to breed every year once they reach maturity (O'shea et al. 2010). There was only one breeding female subsequently recorded as not showing signs of breeding at Hollingbury (*c*. 1% of all females ever recorded) and 15 at Crundale (*c*. 6.6% of all females ever recorded). There is moreover a unidirectional error in the distinction between U and B, and many of those observations are on dates when diagnosis of breeding may be uncertain. Our data thus support the assumption that all bats found to breed at least once were breeders. Models in which female survival and recapture rates were state dependent and/or time dependent as well as constant over both time and state were run in MARK. Transition probabilities were set to be constant over time but dependent on state.

We then used POPAN models in MARK to estimate female abundance at both sites. POPAN estimates three parameters: survival rates (*φ*), recapture probabilities (*p*), probabilities of entry into the population (*pent*), and yield population abundance as a derived parameter. Here, we were only interested in the time-dependent abundance at both sites, we thus ran models where *φ* and *p* were time dependent and independent, and where *pent* was time dependent. This was used alongside an independently derived index of population size at Crundale; emergence counts undertaken for the National Bat Monitoring Programme (NBMP; Bat Conservation Trust 2013).

All models, multistate and POPAN, were compared using Akaike's information criterion for small datasets (AICc). The model with the smallest AICc and those within 2 AICc of it were considered the best fit for the data (Burnham and Anderson 2002).

We finally built a simple female-only stochastic individual-based model (IBM) to project bat population dynamics over time and illustrate the consequences of variation in site-specific demographic rates with three stages: juvenile, immature and breeder. The initial population size was set at 10 individuals in each class, and the time step was 1 year. Juveniles could only remain so for 1 year and at the next year would become immature, breeder, or die; immature bats could remain in that category at the next time-step, or become breeders or die; breeders could only remain breeders or die. State-specific survival rates and transition probabilities between states were taken from observed site-specific rates reported in the main text. Only females in the breeder category could reproduce; we assumed a conservative estimate of 1 pup per female (0.5 females per females). For each population, 1000 simulations of 10 years were run. The average lambda across 10 years was then calculated across 1000 simulations.

## Results

The complete data comprised 749 individual capture and recapture events across a 25-year period. Multiple capture occasions within years were combined into a single annual description per site. Although males were excluded from analyses, we do not anticipate that this should affect the assessment of FCS, because the Serotine bat is likely to be polygynous or promiscuous in common with other European vespertilionids (Crichton and Krutzsch 2000). Thus, productivity should not be diminished because of male scarcity, and knowledge of male demography is not necessary for the prediction of FCS.

Hollingbury was represented by 12 years of data (1989, 1996, 1997, 1999, 2002–2007, 2010, and 2011) describing 102 females. These were recaptured a mean of 1.31 times (SD = 0.58) although 76 individuals (74.5%) were never recaptured, and no bat was ever recaptured more than twice. The average female AFL was 52.7 mm (SD = 1.3, *n *= 70; Fig. S1). Only 10 adults were of known age (marked as juveniles). At this site, 30.8% of all capture events were of individuals classed as U.

Ringing was more continuous at Crundale (all years except 1992 and 1995) describing 228 females which were recaptured a mean of 1.9 times (SD = 1.19), and although 114 individuals (50%) were never recaptured, some were recaptured up to 6 times. There were 42 adults of known age. At this site, 37.7% of all capture events were of individuals classed as U. Emergence counts conducted at Crundale vary annually and suggest that this roost is similar in occupancy to the average for this species in England (NBMP; BCT 2013). The average AFL at Crundale was significantly larger than at Hollingbury (*t*-test, *P *< 0.05) at 53.3 mm (SD = 1.3, *n *= 159; Fig. S1).

### Demography

**Figure 1:**
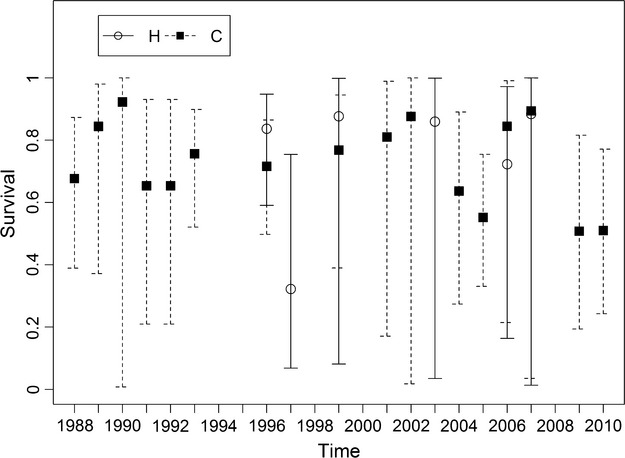
Time-dependent female Serotine bat survival at both sites. Error bars are 95% confidence interval. Estimates are from the models in which survival was a function of time that have the lowest AICc (#7 for Hollingbury and #5 for Crundale).

Three multistate MARK models plausibly fitted the data for the population at Hollingbury (Table1), but only one plausible model was found for the population at Crundale (Table2). At Hollingbury, the top model contained survival as state dependent and the second and third best as constant over time and state. At Crundale, survival was found to be dependent on state. MARK was unable to estimate all annual survival rates for both sites (Fig.1). While associated with large uncertainty, average annual female survival seemed to be consistently higher at Hollingbury. At both sites, breeders had a lower survival rate than bats in the unknown state (Table3). Juveniles showed a similar survival to breeding adults at both sites. As expected, survival rates varied annually, and between sites (Fig.1), but did not appear to show any synchronization despite being at similar altitudes and only 87 km apart.

**Table 1:** Model selection for the Hollingbury population. All models contain transition probabilities *ψ* that are state dependent. ‘npar’ is the number of parameters in each model.

No.	Model	npar	AICc	ΔAICc	Weight	Deviance
1	*S*(˜state) *p*(˜state)	12	271.668	0.000	0.296	155.304
2	*S*(˜1) *p*(˜1)	9	271.852	0.184	0.270	162.860
3	*S*(˜1) *p*(˜state)	10	272.160	0.492	0.231	160.758
4	*S*(˜state) *p*(˜1)	11	273.690	2.022	0.108	159.832
5	*S*(˜1) *p*(˜time)	19	274.690	3.022	0.065	139.274
6	*S*(˜state) *p*(˜time)	21	276.198	4.530	0.031	134.799
7	*S*(˜time) *p*(˜1)	19	287.144	15.476	0.000	151.727
8	*S*(˜time) *p*(˜state)	20	289.039	17.371	0.000	150.664
9	*S*(˜1) *p*(˜state^*^time)	30	293.313	21.645	0.000	121.385
10	*S*(˜state) *p*(˜state^*^time)	32	294.690	23.021	0.000	115.045
11	*S*(˜time) *p*(˜time)	29	303.821	32.153	0.000	135.612
12	*S*(˜time) *p*(˜state^*^time)	40	333.330	61.662	0.000	118.530
13	*S*(˜state^*^time) *p*(˜1)	41	343.243	71.575	0.000	123.491
14	*S*(˜state^*^time) *p*(˜state)	42	347.972	76.304	0.000	123.128
15	*S*(˜state^*^time) *p*(˜time)	51	384.805	113.137	0.000	106.610
16	*S*(˜state^*^time) *p*(˜state^*^time)	62	464.223	192.555	0.000	94.738

**Table 2:** Model selection for the Crundale population. All models contain transition probabilities *ψ* that are state dependent. ‘npar’ is the number of parameters in each model.

No.	Model	npar	AICc	ΔAICc	Weight	Deviance
1	*S*(˜state) *p*(˜state^*^time)	54	2655.647	0.000	0.831	2057.282
2	*S*(˜1) *p*(˜state^*^time)	52	2658.827	3.180	0.169	2065.724
3	*S*(˜state) *p*(˜time)	32	2682.174	26.527	0.000	2138.686
4	*S*(˜1) *p*(˜time)	30	2686.170	30.523	0.000	2147.362
5	*S*(˜time) *p*(˜state^*^time)	73	2695.416	39.770	0.000	2044.008
6	*S*(˜time) *p*(˜time)	51	2715.906	60.259	0.000	2125.412
7	*S*(˜state) *p*(˜state)	12	2769.556	113.909	0.000	2270.784
8	*S*(˜state) *p*(˜1)	11	2770.219	114.572	0.000	2273.567
9	*S*(˜1) *p*(˜state)	10	2771.508	115.861	0.000	2276.966
10	*S*(˜1) *p*(˜1)	9	2773.304	117.658	0.000	2280.862
11	*S*(˜time) *p*(˜state)	31	2776.732	121.085	0.000	2235.590
12	*S*(˜time) *p*(˜1)	30	2781.529	125.882	0.000	2242.721
13	*S*(˜state^*^time) *p*(˜state^*^time)	117	2801.908	146.261	0.000	2001.866
14	*S*(˜state^*^time) *p*(˜time)	95	2809.102	153.455	0.000	2088.455
15	*S*(˜state^*^time) *p*(˜state)	75	2860.481	204.834	0.000	2203.146
16	*S*(˜state^*^time) *p*(˜1)	74	2861.469	205.823	0.000	2207.106

**Table 3:** Demographic rates for female Serotine bats at Hollingbury and Crundale.

Pop	Parameter	Mean	SE	95% CI
H	*S* overall	0.87	0.03	0.80–0.92
	*S* juvenile	0.80	0.12	0.47–0.95
	*S* unknown/intermediate	0.93	0.04	0.82–0.98
	*S* breeder	0.77	0.07	0.60–0.88
	*Ψ*_J→U_	0.42	0.28	0.07–0.87
	*Ψ*_J→B_	0.58	0.28	0.13–0.93
	*Ψ*_U→U_	0.88	0.06	0.73–0.95
	*Ψ*_U→B_	0.12	0.06	0.05–0.27
C	*S* overall	0.81	0.02	0.77–0.84
	*S* juvenile	0.72	0.08	0.53–0.86
	*S* unknown/intermediate	0.88	0.03	0.81–0.93
	*S* breeder	0.75	0.03	0.68–0.80
	*Ψ*_J→U_	1	–	–
	*Ψ*_J→B_	0	–	–
	*Ψ*_U→U_	0.76	0.04	0.69–0.82
	*Ψ*_U→B_	0.24	0.04	0.18–0.31

**Figure 2:**
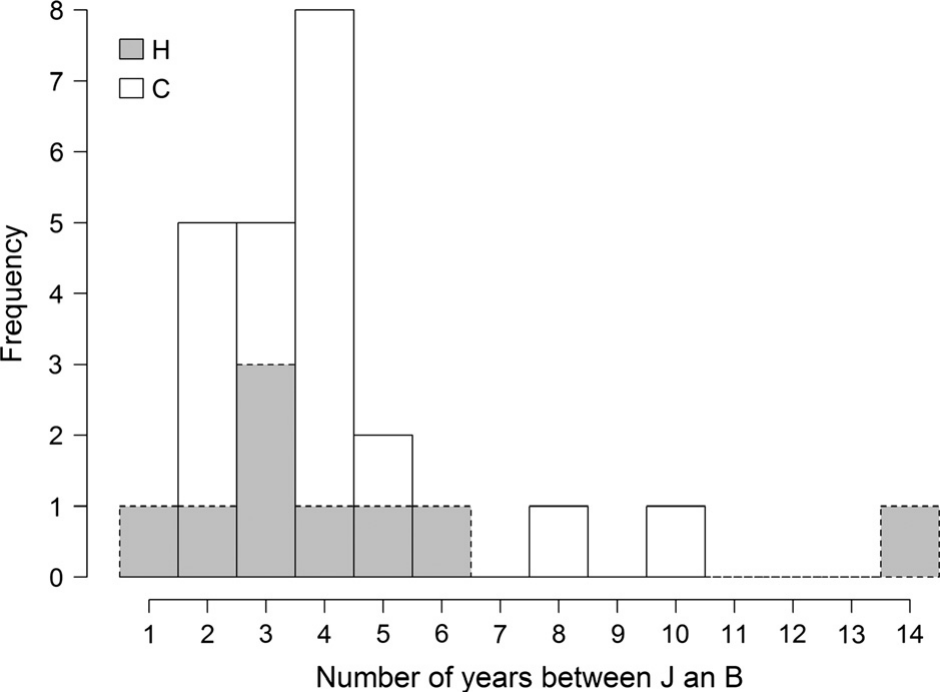
Observed frequency of reproductive lag in female Serotine bats at Hollingbury and Crundale. There were 9 females at Hollingbury and 23 at Crundale for which transition between states J and B could be observed.

Direct transition probabilities between states could only be fully estimated for Hollingbury (Table3) although some of the estimates for this site were poor (e.g., transition J→U:J→B) due to limited data and have very wide confidence intervals. For example, the data included a single instance of a juvenile apparently maturing within a year at Hollingbury (Fig.2), which produced the spurious estimate for this transition. This observation is potentially erroneous (e.g., the bat's age may have been misclassified on first capture) or was exceptional (e.g., its development was so precocious that it had an additional month of development before its first winter); we did not censor the observation because it was not demonstrably wrong. At both sites, females in the unknown stage were more likely to remain in that stage the next year than transition into breeders.

**Figure 3:**
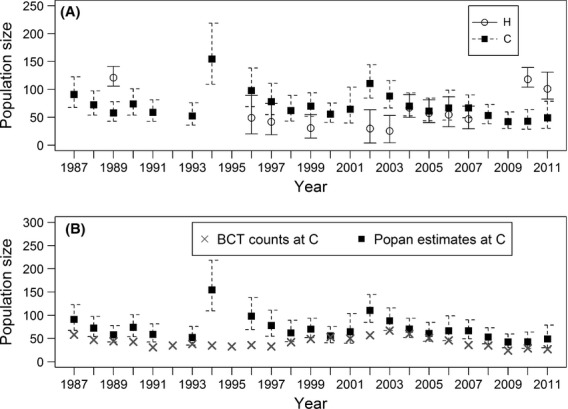
(A) POPAN estimated abundance at both sites and (B) observed emergence counts of bats at site C (Bat Conservation Trust) alongside modeled POPAN estimates. Error bars are 95% confidence intervals. H= Hollingbury; C  =  Crundale.

For both sites, POPAN yielded one unequivocal best model (see Table S1 in Supporting Information). Estimated abundance varied over time (Fig.3), but both populations were stable (observed growth rate for Hollingbury: *λ *= 1.06 ± 0.7; Crundale: *λ *= 1.04 ± 0.5). The independent counts of emerging bats at Crundale also indicated a similar trend (Fig.3).

The breeding histories of recaptured bats were investigated and the observed prereproductive delay of females appeared to be different between the two sites (Fig.2) with the median number of years for individuals to transition J→B was 3 at Hollingbury and 4 at Crundale. The longest observed time for an individual to transit between J and B was 14 years at Hollingbury and 10 at Crundale although we cannot be sure that breeding had not started before this and the bat had either eluded capture in previous years or been mistakenly described. The median time observed between bats classed as J and U was 1 year at both sites (Hollingbury: *n *= 2; Crundale: *n *= 34).

### Population dynamics modeling

**Figure 4:**
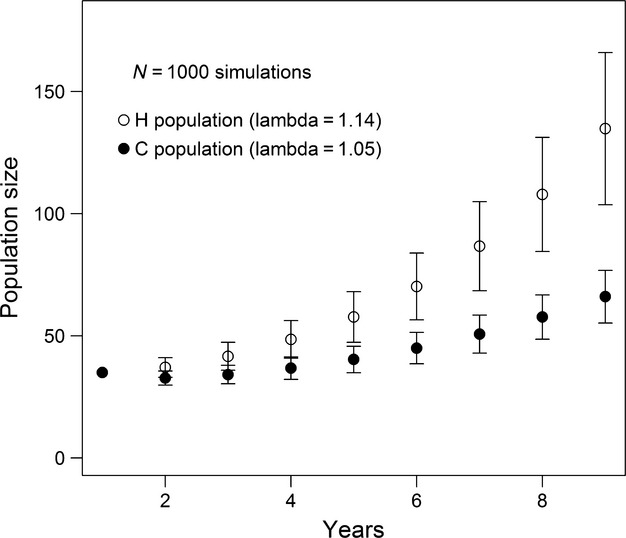
Average modeled population size at site H and C. Error bars represent the standard deviation over 1000 simulations. The initial population size was 30 individuals, distributed evenly in the juvenile, immature, and breeder classes.

The IBM produced distinct trajectories for the two populations. We found the average modeled annual growth rate (*λ*) for Hollingbury was 1.15 (SD = 0.03) compared with 1.05 (SD = 0.02) at Crundale (see Fig.4). As the estimates of abundance for both sites and the independent estimates from the roost emergence counts for Crundale (Fig.3) did not suggest any net increase in the number of bats at these sites, our initial expectation was that our transition probability from U to B may be overestimated.

## Discussion

Here, we provide the first quantitative description of temporal heterogeneity in demographic rates of the Serotine bat at two sites in England with a long history of occupation. We described survivorship for three classes of female and explored the transition rates between them. Our results confirmed the presence of an immature stage for female Serotines in England. We combined these rates to illustrate site-specific population dynamics and marginal population growth.

Survivorship of apparently adult female Serotines was high, which is expected for a member of a taxon renowned for its relative longevity (Hutson 2008). Survival rates were similar to those reported for the American congener *E. fuscus* (O'shea et al. 2011), as well as other recent studies of temperate insectivorous vespertilionids in Europe (Boyd and Stebbings 1989; Sendor and Simon 2003; Schorcht et al. 2009; Papadatou et al. 2011, 2012) and North America (Frick et al. 2010). However, while O'shea et al. (2011) reported rates for a congener in the middle of its transcontinental distribution (at 40°N), we did not expect *E. serotinus* to sustain similar survival at 51°N (i.e., within 150 km of the cold edge of its range). Adult survivorship appears insensitive to latitude and a decline in other demographic rates must be responsible for the marginal dynamic expected for populations at a range edge.

In common with other temperate European bat species (Ransome 1990; Sendor and Simon 2003; Schaub et al. 2007), juvenile survival was lower than adult's, although unlike previous studies where the difference was substantial (Sendor and Simon 2003), juvenile survivorship here was comparable with mature females and only distinct from the unknown class. The availability of Serotine roosts in SE England may be limiting, reducing the opportunity for juveniles to disperse or otherwise spend time away from the nursery site, inflating their relative recapture rate compared with studies in less constrained landscapes. Alternatively, despite comparatively low annual recapture rates, the unusual length of the unknown state allows more independent recapture opportunities increasing their comparative survivorship. Finally, by separating the apparently adult bats into two classes, we refined the relative contributions of these functionally distinct stages to overall adult survivorship and improve the quality of the complete analysis (Pardo et al. 2013).

We confirmed the presence of an immature stage of female Serotines. Labeled unknown, this class was most likely to comprise immature bats otherwise indistinguishable from mature females for most of the year. These experience lower mortality than their breeding conspecifics, probably reflecting their combination of experience at exploiting resources (foraging and roosts) without bearing the annual risks of reproduction, and implying that breeding produces a distinct penalty to survivorship (Clutton-Brock et al. 1989; Koivula et al. 2003). Other possible members of this class were bats caught on dates when detection of breeding is difficult (during May, although variable from year to year), reproductively senescent bats, or mature bats not breeding that year (because they are unmated, failed to initiate a pregnancy, or lost it before it could be detected). While creating a category as uncertain as Unknowns does weaken inference in this study, it was required for this analysis; it is difficult to assign either an age or a breeding status to some bats, because it is currently impossible to accurately age adult bats in the hand without a mark, and some of the traditional characters describing reproduction can be ambiguous (Racey 1974). Moreover, some years are only represented by data sampled on problematic dates, limiting our ability to accurately classify the members of our unknown class. We suggest that the majority of the unknown class was made of immature bats undergoing a prolonged prereproductive delay because of (1) the evidence produced by the individual breeding histories, (2) the likelihood that individuals will remain as Unknowns for more than a year, (3) the low probability that this represents reproductive senescence (Greiner et al. 2014), and (4) the expectation of continuous breeding by mature bats (O'shea et al. 2010). However, consistent differences in the capture methods used at each site may have biased the recapture of the unknown class (e.g., the use of the roof void for captures at Hollingbury may be restricted to pups, their attendant mothers and juveniles, with other classes in the fabric of the roof), which may explain differences in the site-specific mean annual recapture probabilities for this class. In addition, more general social processes in bat communities may reduce the likelihood that immature bats or inexperienced mothers use either of these known and established nursery sites (e.g., they may preferentially use other roosts in their community's spatially extended network), which would explain the higher recapture rates for Breeding bats. It was also possible that the prereproductive delay was itself temporally and spatially variable and dependent upon achieving a threshold body condition or experience (e.g., Speakman and Racey 1986).

Although we found little evidence of large differences in individual demographic rates between our sites, such spatial variation of population processes had been demonstrated in other studies (e.g., Zahn 1999; O'shea et al. 2010, 2011) and was expected. Our IBM illustrated how small, nonsignificant trends across multiple rates can combine to produce substantial differences in population trajectories and predictions of FCS. We suggest such small, difficult to detect, but functionally significant differences in individual demographic rates should not be assumed to represent negligible differences in predictions of FCS. However, the variation of demographic rates (geographic scale and amount) is likely to be hard to predict. Not only will the composition and quality of the foraging landscapes differ between any two communities, but also neighboring nursery roosts (even if close) are likely to have distinct ecophysical properties (e.g., thermal gain and buffering) which may affect population processes.

Our simple, illustrative, stochastic IBM also demonstrated that while site-specific combinations of demographic parameters lead to marginal population growth across the period of study, it could also lead to reductions in community size across shorter timescales, and for the first time, we can suggest, with quantitative evidence, that bat populations may be relatively small, marginal and make a limited contribution to local FCS, even at sites with a long history of occupation (such as Crundale). The persistence of bats at a site may have more to do with their behavior (philopatry) and longevity, rather than the vigor of their populations. The generally poor performance of at least one of our simulated population may be a consequence of the geography of the sites in this study, close to a range edge where the persistence of populations may never be better than marginal. Alternatively, it may be a more general feature of bat ecology in Britain, and we note that no species of vespertilionid show significant positive population trends in the National Bat Monitoring Programme using roost count data (NBMP; Bat Conservation Trust 2013) and that 13 of the 17 breeding species of bat in England are at or close to their range edges (Dietz et al. 2009).

In this study, we have attempted to derive the maximum possible benefit from an amateur dataset that was never designed for the analysis we have undertaken. This has inevitably resulted in compromises in analytical approach and sometimes weaknesses in inference. Notwithstanding these limitations, amateur studies are a key source of data that helps describe the dynamics of a species of conservation concern and may help stimulate the development of an improved evidence base for the effective management of bats. Similarly, while the power of this analysis does not approach those reported for other taxa (e.g., birds), the significance of the collection and reporting of data such as that used here is determined by the paucity of similar studies on bats and the requirement for robust and practicable tools to discriminate source and sink roosts for FCS. As we identify in the introduction, the development of both methods and robust demographic parameters to help manage bats effectively and efficiently is now timely when their populations are expected to be under increasing pressure.

## Conclusion

This work has implications for the conservation and management of bat species in Europe, which is currently based on a poor understanding and prediction of population dynamics in any given scenario (i.e., FCS). Without robust estimates of demographic rates and an understanding of their impact on bat population dynamics, decision-making for the conservation and management of bat populations is unlikely to be optimal. At present, under current environmental conditions and associated threats, there is a pressing requirement to distinguish between sites hosting positive rates of population growth (source sites) and those of marginal or negative growth (sink sites); for example, day-to-day licensing operations require this information to ensure that the mitigation effort used to diminish disruptive development is proportionate, and research workers need to know the context of any study roosts. Our work demonstrates that we can expect Serotine bat populations to have an overall high survival rates but that adults tend to experience a lengthy prereproductive delay. There is variation between life histories of bats at different roosts, and small differences in vital rates can produce vastly divergent population trajectories.

## Data Accessibility

1. Capture–mark–recapture raw files: available from the Dryad Digital Repository: http://doi:10.5061/dryad.6kp2j
2. R script for individual-based model: available from the Dryad Digital Repository: http://doi:10.5061/dryad.6kp2j
3. R scripts and MARK files for capture–mark–recapture analysis: available from the Dryad Digital Repository: http://doi:10.5061/dryad.6kp2j

## Highlights

1. Knowledge of roost sinks and sources is necessary for the successful mitigation of development
2. We describe temporal variation in demographic rates of the Serotine bat
3. We quantify the prereproductive delay in the English Serotine
4. We identify distinct roost demography which yields divergent population trajectories

## Supporting Information

Table S1 Model selection for POPAN models used to estimate abundance at both sites.

Figure S1 Adult Forearm Length of bats of known age for the two sites.
