# Supplementary material for: Demographic variation in the U.K. serotine bat: filling gaps in knowledge for management
Source: Ecol Evol. 2014 Sep 17;4(19):3820–9. doi: 10.1002/ece3.1174 (PMC4301045; doi:10.1002/ece3.1174)
Supplement: Table S1 — Model selection for POPAN models used to estimate abundance at both sites. [file ece30004-3820-sd1.docx]

**Table S1.**

**Model selection for POPAN models used to estimate abundance at both sites.** Survival rate is denoted by *Φ* and recapture probability by *p.* For all models the probability of entry into the population *pent* was time-dependent.

| **model** | **npar** | **AICc** | **ΔAICc** |
| --- | --- | --- | --- |
| **H** |  |  |  |
| *Φ*(~1) *p*(~1) | 14 | 299.14 | 0 |
| *Φ*(~1) *p*(~time) | 25 | 309.67 | 10.5 |
| *Φ*(~1time *p*(~1) | 24 | 314.11 | 14.96 |
| *Φ*(~time) *p*(~time) | 35 | 339.33 | 40.19 |
| **C** |  |  |  |
| *Φ*(~1) *p*(~time) | 47 | 1267.12 | 0 |
| *Φ*(~time) *p*(~time) | 68 | 1302.31 | 35.17 |
| *Φ*(~1) *p*(~1) | 25 | 1327.97 | 60.83 |
| *Φ*(~time) *p*(~1) | 46 | 1337.7 | 70.56 |
