# Supplementary figures and images for: Demographic variation in the U.K. serotine bat: filling gaps in knowledge for management
Source: Ecol Evol. 2014 Sep 17;4(19):3820–9. doi: 10.1002/ece3.1174 (PMC4301045; doi:10.1002/ece3.1174)

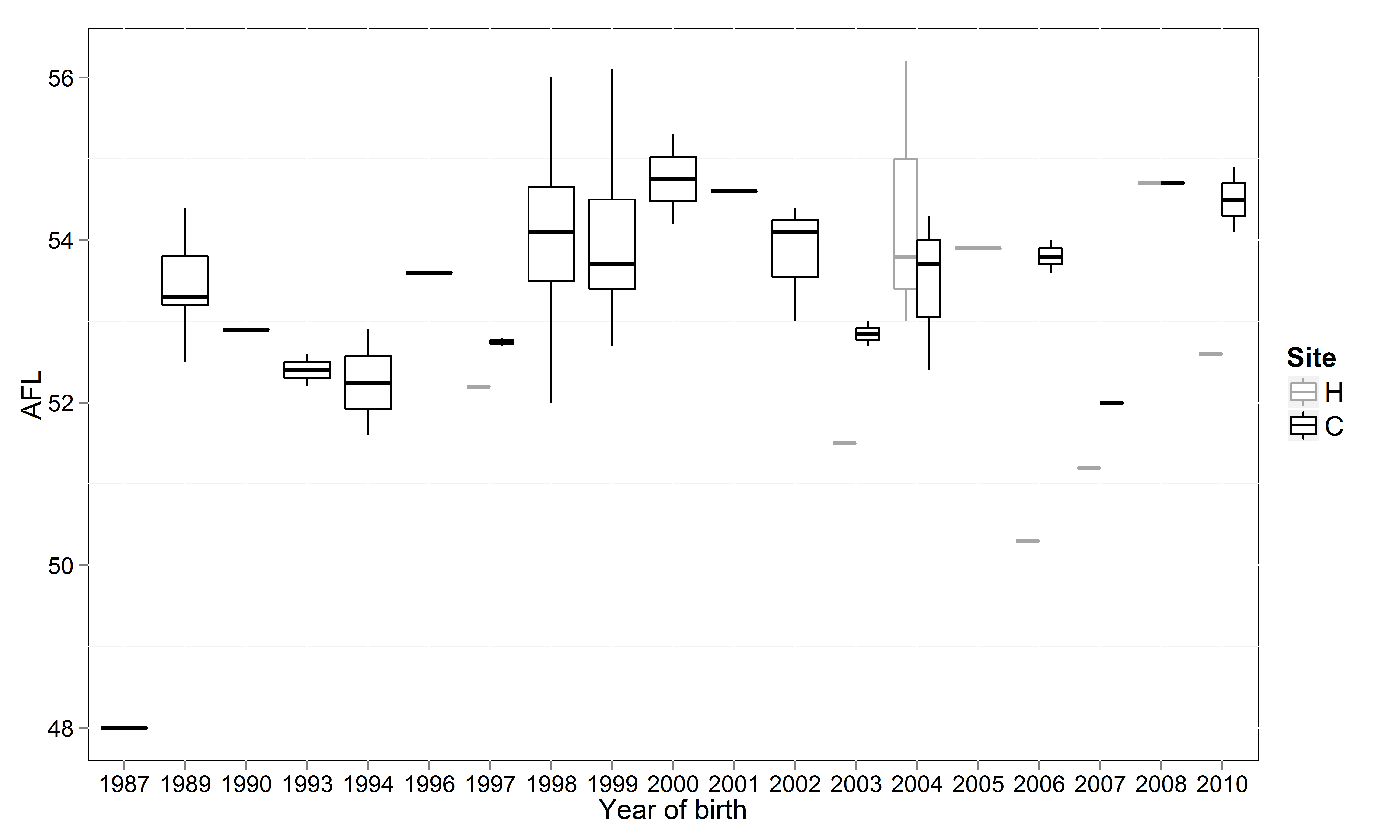

Supplement: Figure S1 — Adult Forearm Length of bats of known age for the two sites. [file ece30004-3820-sd2.jpg]
